# Sex matters for the enhancement of cognitive training with transcranial direct current stimulation (tDCS)

**DOI:** 10.1186/s13293-023-00561-4

**Published:** 2023-11-02

**Authors:** Simone Weller, Birgit Derntl, Christian Plewnia

**Affiliations:** 1 Department of Psychiatry and Psychotherapy, Neurophysiology and Interventional Neuropsychiatry, University of Tübingen, Calwerstraße 14, 72076 Tübingen, Germany; 2 German Center for Mental Health (DZPG), partner site Tübingen, Germany; 3 Department of Psychiatry and Psychotherapy, Innovative Neuroimaging, University of Tübingen, Calwerstraße 14, 72076 Tübingen, Germany

**Keywords:** Brain stimulation, Cognitive control, Cognitive enhancement, Sex differences, Biological sex, Prefrontal cortex, Transcranial direct current stimulation, Neuropsychiatry

## Abstract

**Background:**

Transcranial direct current stimulation (tDCS) can influence brain network activity and associated cognitive and behavioural functions. In addition to the extensive variety in stimulation parameters, numerous biological factors drive these effects, however these are yet poorly understood. Here, we investigate one of the major biological factors by focusing on sex-dependent effects of tDCS on a challenging cognitive control task (*adaptive paced auditory serial addition task* [PASAT]) in healthy humans.

**Methods:**

This sex-specific re-analysis was performed on data of 163 subjects who underwent a 2-week cognitive control training (6 sessions in total). Subjects received either verum (anodal/cathodal) or sham tDCS. Electrodes were placed over the left or right dorsolateral prefrontal cortex and the respective contralateral deltoid muscle. Cognitive control was measured as performance in the PASAT and was analysed in respect to stimulation conditions (sham, anodal, cathodal) and sex.

**Results:**

Regardless of stimulation condition, performance gains between the sexes were higher in females compared to males (*p* = 0.0038). Female’s performance during anodal tDCS exceeded male’s (*p* = 0.0070), yet no effects were found for cathodal or sham tDCS. Moreover, in females we found a superior effect for anodal tDCS over sham stimulation (f_anodal_: *p* = 0.0354; f_cathodal_: *p* = 0.6181), but no such effect in males (m_anodal_: *p* = 0.6882; m_cathodal_: *p* = 0.4822).

**Conclusions:**

This study highlights the relevance of biological sex for the effects of tDCS on cognitive training. Thus, an increased attention to biological sex is advisable in future brain stimulation research to highlight and in consequence better understand potentially underlying sex-specific mechanisms. Considering biological sex will further advance customisation and individualisation of tDCS interventions.

*Trial registration* ClinicalTrials.gov, NCT04108663.

**Supplementary Information:**

The online version contains supplementary material available at 10.1186/s13293-023-00561-4.

## Highlights

- This study provides evidence that tDCS affects females and males differently: females, compared to males, show higher performance gains in a demanding cognitive control task when tDCS is applied concurrently to the task.
- The performance altering effects of tDCS in females were observable for anodal, yet not cathodal stimulation. In males, we did not detect any differences in performance, suggesting that tDCS affects females and males differently and that sex-specific customisation can prove to enhance stimulation efficacy even further.
- Our study highlights that biological sex needs to be taken into account in order to further personalise and optimise the application of tDCS in humans.

**Supplementary Information:**

The online version contains supplementary material available at 10.1186/s13293-023-00561-4.

## Plain language summary

In previous studies, brain stimulation techniques like transcranial direct current stimulation (tDCS) have been shown to support cognitive trainings. However, these effects are rather small and vary between people. A key factor of variability is the biological sex. Hence, in this study we were interested in whether the effects of tDCS differ between females and males. To answer this research question, we analysed the data of 163 human subjects who underwent a 2-week cognitive control training program, which incorporates a challenging cognitive task (the *adaptive paced auditory serial addition task* [PASAT]). During the PASAT, subjects have to solve a stressful calculation exercise. Concurrently to solving this task, the subjects received either real (further divided into anodal [= enhancing] and cathodal [= inhibiting]) or placebo tDCS. We found that females had greater performance gains in the task than males, regardless of the type of tDCS they received. Furthermore, females performed particularly well when they received anodal tDCS, but there were no significant effects for cathodal or placebo tDCS. For males, we did not find any significant benefits of tDCS. These findings highlight the importance of considering biological sex in future brain stimulation research and suggest that biological sex is an important component to consider when studying the effects of tDCS. By paying more attention to this factor, researchers can better understand how tDCS works and develop more effective and personalised interventions.

**Supplementary Information:**

The online version contains supplementary material available at 10.1186/s13293-023-00561-4.

## Background

Transcranial brain stimulation techniques, particularly *transcranial direct current stimulation* (tDCS), have proven to modulate cognitive processes both in healthy as well as patients diagnosed with mental disorders [1–5]. To modify cortical activity via tDCS, electrodes are placed on the scalp with low electrical currents being routed through them. Without evoking action potentials itself, the stimulation is capable to shift the resting membrane potential which in turn affects the resulting neuronal response, i.e. the likelihood of action potentials to occur [6]. In conventional stimulation protocols the anode enhances the neuronal response of the target area, while the cathode reduces cortical excitability at the macroscale level. This property of tDCS has been used to elicit changes in cortical excitability that can last several hours, to modulate cognitive performance in a number of ways, and even to reduce the symptoms of neurological or mental disorders [7].

In a recent study, we systematically analysed the most common stimulation parameters and were able to show that anodal tDCS with an intensity of 1 mA to the left dorsolateral prefrontal cortex (dlPFC) supports cognitive control (CC) processes in healthy humans, while other tDCS configurations did not yield similar results [8]. These cognitive control processes are needed to uphold effective and goal-directed behaviour [9–12], which is required to perform well in the challenging task. Effectiveness of tDCS relies on a plethora of factors. This includes electrode setup (size, shape, orientation) and stimulation polarity as well as brain and head morphology, brain state, pre-existing disorders, usage of psychotropic drugs, hormonal states, age, and sex. The variability in the factors that influence tDCS still limits a systematic use in clinical settings. Not alone the multitude of parameters but also the complex interaction between individual psychological, anatomical, and physiological characteristics with the current flow shape the direction and magnitude of effects. Therefore, it is not surprising that biological sex has already been discussed as a critical factor that contributes to the individual variability of the effects, yet the significance of biological sex for the scientific and clinical use of tDCS remains unclear and an increasing amount of empirical studies reporting sex to be an important variable reinforce this notion [13].

Sex differences include morphological and structural variations [14–16] such as overall head size, larger brain volumes (up to 10%) for males in cerebrum, cerebellum, cerebrospinal fluid, intracranial volume, and deviating tissue density across various brain regions [17]. Additionally, larger volumes of white matter for several brain regions, most notably the frontal cortex, yet no significant difference for global white matter volume [18], and diverging distributions of cancellous bone in the skull [19]. A recent study in a large sample of 240 subjects has shown the extent to which anatomical parameters of the cortex affect the electrical current distribution caused by tDCS [13]. Notably, current densities at the regions of interest varied considerably between females and males, and the distribution of cerebrospinal fluid and grey matter allowed the prediction of current intensities at the target sites. These findings suggest that the ratio between male and female subjects in a study sample influences the outcome. Consistently, a recent meta-analysis on 61 studies supports the notion that, particularly in healthy females, higher current density and/or charge can enhance response accuracy, and that the higher the percentage of females included in the study, the stronger the effect sizes [20].

In addition to these morphological traits, hormone receptors, neurochemicals and -transmitters, which impact neuronal pathways, brain architecture and behaviour [21–24], are expressed at different rates in distinct brain areas between sexes [25], but also between individuals of the same sex [26]. In females, cyclic fluctuations of sex hormones such as endogenous oestradiol [27] should be taken into account [28–31]. As ovarian hormones are known to influence neurotransmission and neuronal excitability [32, 33], they can thereby affect female’s performance in verbal, spatial, and cognitive tasks across the menstrual cycle [30, 31]. Interestingly, the use of hormonal contraception has been found to further influence brain activity, with some activation patterns rather resembling brain activity in males [34].

In terms of sex differences in regard to tDCS, previous studies have shown different outcomes for males and females in specific brain regions such as the visual cortex [35], motor cortex [36], and in different tasks that focus on, e.g. decision-making [37] or theory of mind [38, 39]. Evidently, biological sex affects tDCS efficacy, thereby contributing to the high inter-subject and inter-study variability [40, 41]. To circumvent this, many studies excluded females and were carried out in study samples only including males, thus heavily biasing previous insight towards a male population.

Hence, within this study we focus on this fundamental characteristic of human biology. We re-analysed the sample of 162 healthy subjects form our previously published data [8] with regard to sex differences. The training gains in a challenging cognitive control task over two weeks were compared between females and males receiving either concurrent anodal, cathodal or sham tDCS.

## Methods

This re-analysis is based on previously published data, therefore, we report the materials and methods in brief. A comprehensive description of the experiments is provided in Weller et al. [8]. The study was approved by the University of Tübingen local ethics committee and executed in accordance with the Declaration of Helsinki.

### Experimental design

#### Subjects

In total, 162 subjects were included in the study (127 females, 35 males). Subjects were aged 18 to 39 years (mean age_f_ = 22.73 years, SD = 3.67 years; mean age_m_: 24.89 years, SD = 4.64 years). We acknowledge biological sex not being binary. We distinguish it from gender identity and are aware that sex and gender need not necessarily align.

Before participation, all subjects gave written informed consent. Potential subjects were only included if they reported no diagnosed mental or neurological disorders in the past, no achromatopsia (colour blindness), no metallic implants or tattoos near electrode sites, consumed less than 10 cigarettes per day, sufficient German skills (minimum CEFR level B), and did not take part in any brain stimulation studies while enrolling in this study. Subjects were discharged from our study, and hence their data not used, if they missed a study visit. As compensation, money or course credits were provided with an additional bonus for the best 12 performers.

#### TDCS procedure

Verum stimulation was applied for 19:10 min, therefore starting and ending shortly before and after the PASAT, respectively. Sham stimulation was applied in two blocks, one before and one after the PASAT, limited to a total of 50 s. The current was applied through a CE-certified direct current stimulator (DC-Stimulator MC, NeuroConn GmbH, Ilmenau, Germany; version 1.3.8) and two rectangular rubber electrodes (5 × 7 cm). The stimulation was applied as either sham stimulation (S) or verum stimulation. For verum stimulation, the following configurations were applied: anodal or cathodal polarity (A/C) with an intensity of either 1 mA or 2 mA, applied to either the left or right dlPFC. The position for the first electrode was determined by the international 10–20 system (F3 for left dlPFC, F4 for right dlPFC), the second electrode was placed over the opposing deltoid muscle. The subject’s skin was prepared with mild abrasive gel (Nuprep Skin Prep Gel, Weaver and Company, Aurora, Colorado) and 70% alcohol, electrode surfaces were coated with conductive electrode paste (Ten20 conductive Neurodiagnostic Electrode Paste, Weaver and Company, Aurora, Colorado) and subsequently attached to the skin with adhesive tape.

#### Experimental groups

The two groups (female and male) were split according to tDCS polarity (A/C) to allow the comparison with sham tDCS. To conserve statistical power and group sizes, we did not split the groups further by intensity and laterality as we did in our previous publication. For an overview on the demographical data, see Table 1.

**Table 1:** Demographic group characteristics

Sex	f	m	Test statistic
*N* subjects	127	35	Not applicable
Age^b^	22.73 (3.67)	24.89 (4.64)	*t*(160) = 2.898, *p* = 0.004*
EHI-Score^b^	0.904 (0.1318)	0.940 (0.0914)	*t*(160) = 1.520, *p* = 0.131
Last math grade^b^	2.29 (1.078)	2.26 (0.954)	*t*(160) = − 0.113, *p* = 0.910
QCM (anxiety)^b^	3.5795 (1.2094)	3.5086 (1.2313)	*t*(160) = − 0.306, *p* = 0.760
QCM (success)^b^	4.0735 (1.2791)	4.3897 (1.2839)	*t*(159) = 1.279, *p* = 0.203
QCM (interest)^b^	3.9925 (1.1751)	4.2457 (1.2603)	*t*(160) = 1.111,* p* = 0.268
QCM (challenge)^b^	5.2165 (0.9206)	5.3571 (0.9301)	*t*(160) = 0.798, *p* = 0.426
RSES^b^	39.09 (5.314)	40.06 (5.263)	*t*(158) = 0.956, *p* = 0.341
Hormonal contraceptive (yes/no)^c^	64/63	Not applicable	*χ*^2^(1) = 0.008,* p* = 0.929
Smoking (yes/no)^a^	15/112	8/27	*p* = 0.087

Means and standard deviations (M(SD)) are shown; if not applicable, the number of subjects belonging to each trait are shown. One female subject in the cathodal group was removed from our analyses, as her performance deviated more than 2 SD from all other subjects, resulting in a total of 162 instead of 163 subjects

*EHI*  Edinburgh Handedness Inventory, *QCM*  Questionnaire on Current Motivation, *RSES*  Rosenberg Self-esteem Scale

^a^Fisher’s exact test

^b^*t*-test

^c^Chi^2^

*p < 0.05

#### Cognitive control training: PASAT (Fig. 1A)

**Fig. 1:**
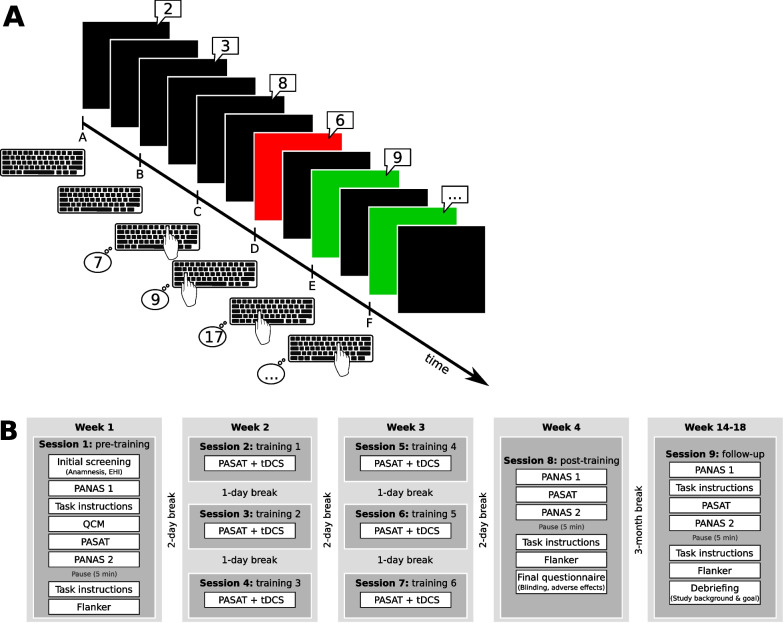
During the PASAT, single digit numbers were presented to each subject. They were asked to sum the current (*n*th) and second-to-last digit (*n*^th−2^): e.g. digits at timepoints C and A, D and B, E and C, and so on. Several correct answers in a row shortened the interval between digit presentations while long answers prolonged it (**A**). Subfigure **B** shows the timeline of the experiment. Figure adapted from Weller et al. [8], reprinted with permission

We used a modified adaptive version of the PASAT. Subjects were seated in front of a computer screen. Over headphones, they heard single digit numbers in random order and were instructed to add the current digit to the digit that preceded it by 2 (*n*^th^ + *n*^th−2^). Responses were given on a keyboard with all possible results printed on it (i.e. the numbers 2 to 18). Subjects were instructed to answer as quickly and correctly as possible. If subjects answered correctly/incorrectly four times in a row, the interval with which the digits were presented was decreased/increased by 0.1 s, resulting in performance-dependent task speed. At the beginning of each session, the interval between digits was 3 s and then adjusted according to performance. Each training session was divided into 3 blocks, 5 min each, with the achieved interval being carried over from block to block. Between each block a 30 s pause was implemented. Subjects were only allowed to give answers with their right index finger.

This form of the *2-back* PASAT [42] was chosen over the standard *1-back* PASAT, where the last digit must be added to the digit directly before it. From our experience, the 1-back PASAT would likely have been too easy for our healthy group and would have culminated in ceiling effects.

#### Study timeline (Fig. 1B)

In total, each subject attended nine sessions. Session one to eight happened within one months’ time (pre-training in week 1, six training sessions in week 2 and 3, post-training in week 4). The last session (follow-up) was conducted three months later. During each session subjects carried out the PASAT, however tDCS was applied only during training sessions. Training sessions alternated with one training-free day.

#### Questionnaires

To assess for right-handedness, only subjects scoring higher than 60 in the Edinburgh Handedness Inventory (EHI) could participate in this study [43]. This was done to minimise possible variability in tDCS response caused by subjects’ handedness [44]. Through the Questionnaire on Current Motivation (QCM), we a priori accounted for overall interest and perceived challenge in the task as this might have subsequently influenced performance [45]. Other anamnestic data such as age and formal education were inquired about in a custom questionnaire. We measured subjects’ self-esteem through a modified Rosenberg Self Esteem Scale (RSES) which allowed to measure self-esteem scores between 10 and 50 [46]. For a summary of the assessed items please refer to Table 1. It is of note, that this RSES utilises a 5-point Likert scale incorporating a middle category of agreement (“neither agree nor disagree”), unlike the original version of the questionnaire which only offers 4 points. This might increase variability of responses or reduce acquiescence bias, and analyses regarding varying numbers of Likert scale points show no difference in external validity [47].

### Statistical analyses

Unless stated differently, threshold for type I error was set to 5% and all tests refer to two-tailed tests. R version 3.5.1 to 4.0.2 [48] with packages nlme [49] as well as regehlper [50] and IBM SPSS Statistics Software version 24 [51] were used for all analyses.

#### Cognitive control training: effects of tDCS between sexes

To assess performance, the number of correct trials within each training session (*n*_corr_) was calculated. This was done as the PASAT was limited by time (15 min raw PASAT), hence subjects were able to solve as many calculations during a session as their abilities allowed. As faster digit presentations were a result of better performance (i.e. higher count of *n*_corr_), this variable was chosen as a comparator between the study groups.

Since pre-training performance (*n*_corr(pre)_) might prove to be an indicator for overall performance, this value was compared separately between the female and male group. For this, we used a mixed-effects ANOVA with the within-subject factor *n*_corr(pre)_ and between-subjects factor sex.

Next, the training sessions (session two to seven) were analysed. For each of the following steps, performance gain measured in one sex was compared to performance gain of the other sex. All this was done in a linear mixed-effects model: sex, time (i.e. session number), and the interaction sex x time were used as fixed effects. Performance (*n*_corr_) was used as the dependent variable, and *n*_corr(pre)_ was included as a regression coefficient. Random effects were measurement timepoint and individual subject (~ *1* + *time* | *subject*). Firstly, subjects were grouped by sex only, regardless of the tDCS intervention (males, pooled: m_P_; females, pooled: f_P_). Secondly, we split the groups by tDCS *polarity* to test for possible polarity-dependent deviations: performance gain of all males from the sham group (m_S_) was compared to the performance gain of all females from the sham group (f_S_). Analogous, the performance gain of the anodal (m_A_ and f_A_) and cathodal (m_C_ and f_C_) groups were compared.

#### Cognitive control training: effects of tDCS within each sex

While the afore-described steps allow for the analysis of effects between the two sexes, they do not answer the question whether any tDCS condition caused effects within each sex. Therefore, we ran a linear mixed-effects model for each sex independently. For this, we compared performance of subjects who had received either anodal or cathodal tDCS to subjects of the same sex who had received sham tDCS (m_A_ and m_C_ compared to m_S_; f_A_ and f_C_ compared to f_S_). Again, *n*_corr_ was used as the dependent variable and *n*_corr(pre)_ was included as a coefficient. Fixed effects were defined as the condition (S/A/C), time (corresponding to session), and the interaction between condition x time. As random effects, measurement timepoint and individual subject (~ *1* + *time* | *subject*) were used.

We refrained from computing non-standardised (*B*) and standardised beta coefficients (*β*), for why measuring effect strength is still a topic of discussion where an optimal roadmap has yet to be developed. This goes in accordance with the reasoning given when the *beta()* function included in R’s reghelper package [52–55] was deprecated. The between-sex analyses were corrected via the Bonferroni–Holm method, as the polarity sample is a subgroup of the pooled sample. Lastly, to look at possible long-term effects, performance gains of the groups were tested against each other via *t*-tests.

#### Questionnaires

The questionnaires were implemented to ensure similar group compositions, comparative analyses between male and female groups were performed using Fisher’s Exact test, *t*-test, Chi^2^ test with each questionnaire’s *outcomes* as dependent variables (Table 1).

## Results

### Sample characteristics

No disparities were found for pre-training performance between females and males, showing that subjects started the study at similar performance levels: *F*(1, 160) = 1.809, *p* = 0.180, *η*^2^ = 0.011. The distribution of females menstruating during the training phase did not differ between the groups split by tDCS polarity (S/A/C; Fisher’s exact *p* = 0.659). While there was a significant age difference (males being older by an average of 2 years), there were no differences for any of the other descriptive factors; please refer to Table 1 for a comprehensive overview.

### Cognitive control training: between sex effects

**Fig. 2:**
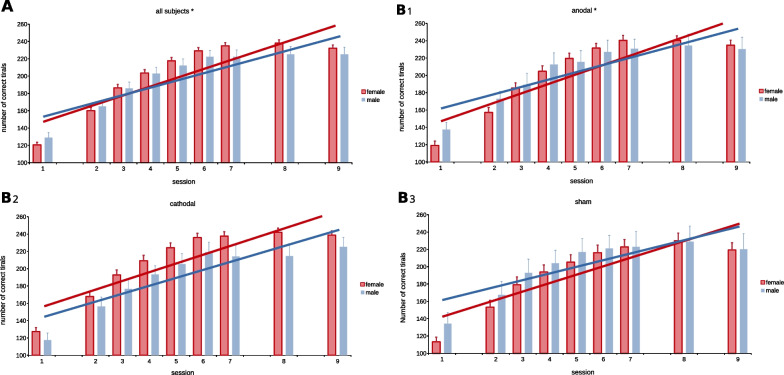
Performance development between the two sexes. Measurement of performance is the sum of correct trials per session. Shown here is the performance for **A**: all subjects and **B1**–**B3**: separated by tDCS polarity. For every subfigure, females’ performance gains are compared to males’. Trendlines indicate performance gains over time, with steeper inclines corresponding to higher performance gains. We found a significant effect of sex when analysing all conditions in a single group (**A**), with female’s performance increase surpassing male’s (*p* = 0.0038). For polarity (**B1**–**B3**), we found that females improved significantly over males under anodal conditions (**B1**; *p* = 0.0070), whereas no difference was found under cathodal (**B2**; *p* = 0.3258) or sham condition (**B3**; *p* = 0.2063). *Groups where performance gains differ significantly (p < 0.05)

Figure 2 shows subjects’ performance gains over time. Additional file 1: Table S1 provides the raw *n*_corr_ for all possible groups. The exhaustive statistics for all analyses are provided in Additional file 1: Table S2–S7. Main effects of time and *n*_corr(pre)_ were highly significant in all cases (*p* < 0.001), showing that subjects improved their performance over the course of the training and that pre-training performance was a predictor for further performance gains, with higher pre-training performance correlating with increased performance gains during the subsequent training period. The significant differences in training effects between the two sexes, that we were able to find during the training period, did not persist for post-training or follow-up (post-training: *t*(45.568) = −1.391, *p* = 0.171; follow-up: *t*(158) = 0.841, *p* = 0.402). No difference in baseline performance was found for either analysis (pooled group: *p* = 0.180; anodal group: *p* = 0.105; cathodal group: *p* = 0.320; sham group: *p* = 0.078. Bonferroni–Holm corrected threshold: *p* = 0.025).

#### Analysis of all subjects combined regardless of tDCS parameters (m_P_/f_P_)

Between the two pooled groups, we found an effect of sex, indicating that females exhibited higher training gains compared to males (*p* = 0.0038). The Bonferroni–Holm corrected threshold for significance is 0.0125. Based on this significant general sex difference, we split groups according to the applied polarity. See Additional file 1: Table S2.

#### Analysis of subjects according to stimulation polarity (m_S_/f_S_; m_A_/f_A_; m_C_/f_C_)

Here, we found a significant effect for anodal polarity. As above, females showed higher performance gains than males when anodal tDCS was applied (*p* = 0.0070 with Bonferroni–Holm corrected threshold of 0.0167), however no such sex effect emerged for the sham (*p* = 0.2063 with Bonferroni–Holm corrected threshold of 0.025) or cathodal condition (*p* = 0.3258 with Bonferroni–Holm corrected threshold of 0.05, respectively). See also Additional file 1: Table S3–S5.

### Cognitive control training: within-sex effects

**Fig. 3:**
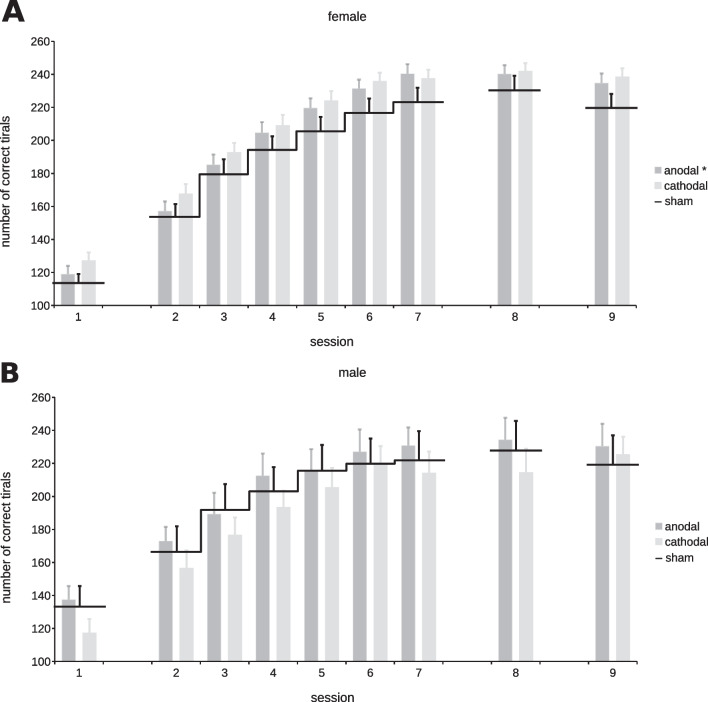
Performance development within each sex. Measurement of performance is the sum of correct trials per session. Shown are the number of correct trials for all females in **A** and males in **B**, split each by tDCS polarity. The performance for each polarity condition (bars) was compared to sham stimulation (black line). We found significant performance gains for females in the anodal group compared to sham (*p* = 0.0354). This effect was not seen in the cathodal group, where the performance increase of cathodal and sham stimulation was of similar magnitude throughout the training phase (*p* = 0.6181). No significant effects were found for either polarity in the male group, as both polarities resulted in similar performance gains compared to sham stimulation (*m*_A_: *p* = 0.6882; *m*_C_: *p* = 0.4822). *Groups where performance gains differ significantly (*p* < 0.05)

While the hierarchical analysis above answers the question whether females and males varied in their performance gains, it does not allow to draw conclusions about performance changes based on tDCS polarity within each sex. Hence, we analysed the two sexes independently thereby exploring possible tDCS effects that are prominent in one sex but absent in the other. Figure 3 shows the number of correct trials per training session within each sex.

#### Male group: polarity (m_S_/m_A_/m_C_)

We found no differences in performance gains for *polarity* between the male verum group compared to the male sham group (m_A_: *p* = 0.6882; m_C_: *p* = 0.4822). See also Additional file 1: Table S6.

#### Female group: polarity (f_S_/f_A_/f_C_)

Assessing for polarity effects in females, we observed a significant effect with females receiving anodal tDCS performing better over the training sessions than the female sham group (*p* = 0.0354). This was not the case for the cathodal group compared to sham (*p* = 0.6181). The effect for the anodal group did not persist throughout post-training (*t*(52.994) = −1.009, *p* = 0.318) or follow-up (*t*(79) = −1.578, *p* = 0.119). See also Additional file 1: Table S7.

## Discussion

In this re-analysis of previously published data, we tested the influence of biological sex on tDCS-supported enhancement of cognitive control training in healthy females and males who underwent a challenging 2-week training paradigm (PASAT). For the whole study group, including all stimulation conditions (anodal, cathodal, sham), we found a larger training benefit in females compared to males over the course of the training phase. More precisely, females had consistently higher training gains compared to males when anodal tDCS (but not cathodal or sham) was applied to the prefrontal cortex during training. Consistently, the comparison of stimulation conditions within sexes demonstrated a beneficial effect of anodal over sham tDCS in females, but no such effect in males. No effects were found in either sex for cathodal over sham tDCS. Thus, our analysis indicates that the enhancement of cognitive control training by anodal tDCS is critically modulated by biological sex, with females being more susceptible for beneficial effects than males. A similar level of performance at baseline and the lack of differential effects in the sham group underline the specificity of this effect: the absence of differences in educational levels and expectation towards the task as assessed via questionnaire indicate comparable performance prerequisites in both sexes, and both groups had similar math abilities and motivation to perform well in the task.

So far, the available data on the interaction between biological sex and tDCS are highly inconsistent with some studies reporting effects only in one sex while being absent, or even opposite, in the other [20, 56, 57]. For example, Meiron et al. showed beneficial effects of anodal tDCS during a verbal n-back task with stimulation of the left dlPFC in their male sample, whereas in females stimulation of the right dlPFC proved to achieve similar positive effects [58]. He et al. found, that while both sexes benefitted from anodal tDCS over the left dlPFC in the Iowa Gambling Task, in females the stimulation effect was more pronounced [59]. In our study, we were able to show that anodal tDCS facilitated training gains in females, but not males.

As diverse as the outcomes of anodal tDCS alone are, so are the results from studies focusing on cathodal tDCS. For example, some research has found excitatory effects of cathodal tDCS over the motor cortex. This effect however, was only prominent for certain current intensities. More strikingly, the magnitude, duration, and direction of these non-linear effects were dependent on stimulation intensity [60]. Another study found that cathodal tDCS increased performance in a cognitive task, instead of degrading performance [61]. These examples alone highlight even more how complex the relationship between tDCS parameters and their potential effects is. While we were not able to observe any effects for cathodal tDCS in our study, it is possible that this resulted from a certain parameter combination we did not analyse, the study group that was included in the experiments, or the task that was done during the stimulation. Lastly, it is not yet clear if or how the magnitude of outcomes from anodal and cathodal tDCS are related.

It seems reasonable to assume that the sex-dependent variability in our study is at least partially explained by anatomical differences for instance in volume of cerebrospinal fluid, skull thickness, gyrus orientation, or the individual location of the dlPFC. It should be noted that the relevance of sex differences in brain architecture for cognitive functions and mental health are still under debate. Two recent analyses based on MRI data [62, 63] coincide that structural differences in brain morphology between males and females exist, but draw contrasting conclusions on their impact: ranging from the differences being trivial (e.g. derived from height and size of the subject) and the brain not being sexually dimorphic [63], to several regions still being significantly distinct even when accounting for overall body morphology [64]. In the end, current status can potentially agree on the “mosaic” hypothesis, indicating that no typical female or typical male brain exists without neglecting frequently reported differences in brain anatomy and function [65]. Based on this variability, it can be assumed that current flow that reaches the cortical area relevant for cognitive control processes differs between females and males [66]—and hence identical tDCS configurations not necessarily lead to comparable results in all sexes. However, some researchers suggests that this variability is more appropriately described by means of these anatomical features than in regard to biological sex differences [13, 67, 68]. Nevertheless, it has also to be considered that inter-individual variability possibly outweighs sex effects [69], though greater variance in brain structure was reported in males than females across the lifespan [16].

Besides anatomical variability, variations in task-specific activation of brain networks exist and reinforce the significance of the task being conducted during the application of tDCS. Sex-specific activation patterns have been found in various cognitive tasks as well as in the processing of emotional information [70–72]. For instance, under specific task conditions, females have been shown to more strongly involve higher-order frontal regions such as the prefrontal cortex which could be further enhanced by tDCS [69, 73]. In another study more pronounced effects of tDCS were visible when tDCS was applied on the hemisphere that was predominantly activated during a specific task [74]. With this evidence we can assume that the beneficial effects of tDCS in females were likely also influenced by the underlying specific activation patterns within the frontal brain regions.

More specifically, and in addition to the aforementioned differences, variability between the sexes in challenging cognitive tasks might be linked with the fact that females and males show different brain activity particularly in response to cognitive stress, which then in turn affects performance during those stressful tasks [75–77]. Therefore, it can be assumed that tDCS shows sex-specific differences of brain activity in a stressful task like the PASAT [78]. More precisely, females have been shown to be more sensitive to negative feedback [79], which is a major component in the PASAT, posing an additional challenge for the executive system. Both workload and cognitive state can influence the efficacy of tDCS. Li et al. showed that effects of anodal tDCS were more pronounced without a concurrent task while the opposite was true for cathodal tDCS, yet, results like these seem to highly depend in the task itself [80, 81]. However, as stress increases workload [82], a consequently higher activity of the dlPFC might be the basis for a higher response to tDCS in females specifically.

Within our sample we found an age difference of approximately 2 years between males and females. Previous studies have shown that age seems to be related to tDCS efficacy. Supposedly, this effect can be related to brain atrophy and that the aging brain, with its accompanying changes in morphology, requires different tDCS parameters to be effective [83]. This data suggests that the higher age difference between groups, the more closely parameters should be inspected and adapted—especially current dose. However, the study also notes that the brain ratio as a measure of brain atrophy, rather than chronological age, plays the larger role in the response to tDCS. As the age difference in our sample is very small and both groups would still fall within the same age cluster (i.e. young adults), we presume that biological sex is the main driving factor for the performance variations we found.

Conclusively, structural and functional anatomy of cognitive control training likely varies between males and females. Indeed, recent studies allow the assumption that individual components of cognitive control may be altered differently in the sexes (yet without systematic advantage) and that these effects depend on the modality of testing and respective parameters [58, 75]. It stands to reason that both sexes employ different strategies when presented with challenging tasks [76, 84, 85] such as the PASAT. The specific strategies and how they can be enhanced by concurrent tDCS, remains elusive so far, but increasing evidence for this theory has been found recently.

A limitation of this retrospective analysis is the unequal distribution of sexes in our sample. While the applied statistical models are robust enough to account for the numerical distribution, the smaller number of male subjects did not allow to analyse the influence of sex on stimulation intensity and laterality. Another challenge is that the hormonal states of individual subjects can influence tDCS and should therefore be monitored thoroughly: oestradiol is known to enhance cortical excitability and should be considered when excitability is modified via tDCS, while at the same time, progesterone can decrease excitability [86]. Higher levels in oestradiol (compared to lower levels) have shown greater neuroplastic responses when tDCS was applied to the frontal cortex, hence suggesting that oestradiol contributes to inter-individual variability in tDCS outcomes [27]. As our data allow to analyse the distribution between females menstruating/not menstruating during the experiment, we found that this distribution was equal. However, we did not collect more specific data on menstrual cycles to determine the exact cycle phase of each female. As oestradiol peaks before ovulation but rises again during the mid-luteal phase, simply comparing females who are menstruating (low levels) with not menstruating females (varying levels) is not enough. Additionally, half of our female sample were using hormonal contraception, thus further influencing sex hormone levels. This needs to be addressed in more detail in further prospective studies, focussing specifically on this research question.

Another aspect to consider is the question whether the observed effects are actually related to biological sex or whether they are mostly correlated to anatomical differences. However, as certain anatomical features and sex heavily correlate with each other, this is more a matter of perspective and phrasing and hence the critical interaction between sex and tDCS intervention outcomes remains.

Finally, in an adult human sample effects of sex can hardly be disentangled from effects of gender and gender roles. Self-concepts and personality traits, such as neuroticism and conscientiousness, that are more expressed in females [87], can influence behaviour and are thought to be influenced by experience, social desirability concerns, and societal norms. We did not assess gender identity, gender norms and gender expression in our participants which should be done in future studies to shed light on how gender and other diversity aspects influence reported results.

In sum, we can conclude that research is picking up on the importance of sex differences in the neuromodulation of the human cortex. With this study we shed further light on the variable impact of tDCS on performance in a cognitive task and whether this is influenced by biological sex. Most likely, sex-related diversities are not binary but lie on a complex spectrum composed of morphological, hormonal, and neurobiological factors. Researchers should harness the knowledge on sex differences to stratify and personalise brain stimulation interventions. Especially in the light of tDCS being a viable tool for the treatment of various illnesses, it is vital to further uncover the (biological) characteristics that have a bearing on tDCS efficacy and hence contribute to the high variability we currently see in the study landscape. By doing so, personalised interventions may prove to surpass standardised paradigms soon.

### Perspective and significance

Non-invasive transcranial brain stimulation is a powerful tool to influence cognitive performance and training. The stimulation effects can be modulated by a multitude of factors one of them is sex/gender. While the results of this study suggest that tDCS works better in females when faced with a challenging cognitive task, we cannot conclude that there are no effects in males. This will require a more focused and sex-based approach. Understanding how sex interacts with tDCS is a critical step on the path to personalised and effective cognitive interventions and treatments.

## Conclusions

Our results are the first to show that beneficial effects of anodal tDCS on cognitive control training are more prominent in females than in males. This supports the notion that biological sex is one of the critical sources of variability in tDCS responses on cognitive training in particular and most likely in neuromodulation in general. Notably, these sex effects are measurable under anodal tDCS, however not under cathodal or sham condition. When comparing tDCS polarities within the sexes, anodal tDCS proved to be beneficial over sham and cathodal tDCS for females, however that was not the case for males. Accordingly, our results clearly point towards a further individualisation of tDCS by recognising biological sex. Further research is required to elucidate the specific interrelations between biological, social and functional characteristics of individuals and stimulation techniques. Based on this, more refined tDCS interventions show a promising perspective to yield optimal results for research and therapy.

### Supplementary Information

**Additional file 1: Table S1.** Training performance as measured by the number of correct trials for all groups. Shown are mean of correct trials and standard deviations in parentheses. **Table S2**. Results from the linear mixed model for all males and females pooled, regardless of tDCS setting. All calculations use the female group (f_P_) as a reference. Number of subjects: *N* = 162. **Table S3.** Results from the linear mixed model for groups organised by tDCS polarity (sham). All calculations use the female group (f_S_) as a reference. Number of subjects: *N* = 43. **Table S4.** Results from the linear mixed model for groups organised by tDCS polarity (anodal). All calculations use the female group (f_A_) as a reference. Number of subjects: *N* = 60. **Table S5.** Results from the linear mixed model for groups organised by tDCS polarity (cathodal). All calculations use the female group (f_C_) as a reference. Number of subjects: *N* = 59. **Table S6.** Results from the linear mixed model from the male group divided by tDCS polarity. All calculations use the sham group (m_S_) as a reference. Number of subjects: *N* = 35. **Table S7.** Results from the linear mixed model from the female group divided by tDCS polarity. All calculations use the sham group (f_S_) as a reference. Number of subjects: *N* = 127.

## Data Availability

The datasets used and/or analysed during the current study are available from the corresponding author on reasonable request.

## References

[1] Shiozawa P, Fregni F, Benseñor IM, Lotufo PA, Berlim MT, Daskalakis JZ (2014). Transcranial direct current stimulation for major depression: an updated systematic review and meta-analysis. Int J Neuropsychopharmacol.

[2] Salehinejad MA, Wischnewski M, Nejati V, Vicario CM, Nitsche MA (2019). Transcranial direct current stimulation in attention-deficit hyperactivity disorder: a meta-analysis of neuropsychological deficits. PLoS ONE.

[3] Moffa AH, Martin D, Alonzo A, Bennabi D, Blumberger DM, Benseñor IM (2020). Efficacy and acceptability of transcranial direct current stimulation (tDCS) for major depressive disorder: an individual patient data meta-analysis. Prog Neuropsychopharmacol Biol Psychiatry.

[4] Boggio PS, Ferrucci R, Rigonatti SP, Covre P, Nitsche M, Pascual-Leone A (2006). Effects of transcranial direct current stimulation on working memory in patients with Parkinson’s disease. J Neurol Sci.

[5] Brunelin J, Mondino M, Gassab L, Haesebaert F, Gaha L, Suaud-Chagny MF (2012). Examining transcranial direct-current stimulation (tDCS) as a treatment for hallucinations in schizophrenia. Am J Psychiatry.

[6] Nitsche MA, Paulus W (2000). Excitability changes induced in the human motor cortex by weak transcranial direct current stimulation. J Physiol.

[7] Nitsche MA, Paulus W (2001). Sustained excitability elevations induced by transcranial DC motor cortex stimulation in humans. Neurology.

[8] Weller S, Nitsche MA, Plewnia C (2020). Enhancing cognitive control training with transcranial direct current stimulation: a systematic parameter study. Brain Stimulat.

[9] Brunoni AR, Vanderhasselt MA (2014). Working memory improvement with non-invasive brain stimulation of the dorsolateral prefrontal cortex: a systematic review and meta-analysis. Brain Cogn.

[10] Birba A, Ibáñez A, Sedeño L, Ferrari J, García AM, Zimerman M (2017). Non-invasive brain stimulation: a new strategy in mild cognitive impairment?. Front Aging Neurosci..

[11] Lawrence BJ, Gasson N, Bucks RS, Troeung L, Loftus AM (2017). Cognitive training and noninvasive brain stimulation for cognition in Parkinson’s disease: a meta-analysis. Neurorehabil Neural Repair.

[12] Sathappan AV, Luber BM, Lisanby SH (2019). The dynamic duo: combining noninvasive brain stimulation with cognitive interventions. Prog Neuropsychopharmacol Biol Psychiatry.

[13] Bhattacharjee S, Kashyap R, Goodwill AM, O’Brien BA, Rapp B, Oishi K (2022). Sex difference in tDCS current mediated by changes in cortical anatomy: a study across young, middle and older adults. Brain Stimulat.

[14] Filmer HL, Ehrhardt SE, Shaw TB, Mattingley JB, Dux PE (2019). The efficacy of transcranial direct current stimulation to prefrontal areas is related to underlying cortical morphology. Neuroimage.

[15] Ritchie SJ, Cox SR, Shen X, Lombardo MV, Reus LM, Alloza C (2018). Sex differences in the adult human brain: evidence from 5216 UK biobank participants. Cereb Cortex N Y N 1991..

[16] Wierenga LM, Doucet GE, Dima D, Agartz I, Aghajani M, Akudjedu TN (2022). Greater male than female variability in regional brain structure across the lifespan. Hum Brain Mapp.

[17] Ruigrok ANV, Salimi-Khorshidi G, Lai MC, Baron-Cohen S, Lombardo MV, Tait RJ (2014). A meta-analysis of sex differences in human brain structure. Neurosci Biobehav Rev.

[18] Bourisly AK, Gejo G, Hayat AA, Alsarraf L, Dashti FM, Paola MD (2017). White matter sexual dimorphism of the adult human brain. Transl Neurosci.

[19] Russell M, Goodman T, Wang Q, Groshong B, Lyeth BG (2014). Gender differences in current received during transcranial electrical stimulation. Front Psychiatry..

[20] Dedoncker J, Brunoni AR, Baeken C, Vanderhasselt MA (2016). A systematic review and meta-analysis of the effects of transcranial direct current stimulation (tDCS) over the dorsolateral prefrontal cortex in healthy and neuropsychiatric samples: influence of stimulation parameters. Brain Stimulat.

[21] Kim MS, Koo H, Han SW, Paulus W, Nitsche MA, Kim YH (2017). Repeated anodal transcranial direct current stimulation induces neural plasticity-associated gene expression in the rat cortex and hippocampus. Restor Neurol Neurosci.

[22] Stagg CJ, Best JG, Stephenson MC, O’Shea J, Wylezinska M, Kincses ZT (2009). Polarity-sensitive modulation of cortical neurotransmitters by transcranial stimulation. J Neurosci.

[23] Knouse MC, McGrath AG, Deutschmann AU, Rich MT, Zallar LJ, Rajadhyaksha AM (2022). Sex differences in the medial prefrontal cortical glutamate system. Biol Sex Differ.

[24] Rehbein E, Hornung J, Sundström Poromaa I, Derntl B (2021). Shaping of the female human brain by sex hormones: a review. Neuroendocrinology.

[25] Vries GJ (1990). Sex differences in neurotransmitter systems. J Neuroendocrinol.

[26] Bixo M, Bäckstrӧm T, Winblad B, Andersson A (1995). Estradiol and testosterone in specific regions of the human female brain in different endocrine states. J Steroid Biochem Mol Biol.

[27] Lee S, Chung SW, Rogasch NC, Thomson CJ, Worsley RN, Kulkarni J (2018). The influence of endogenous estrogen on transcranial direct current stimulation: a preliminary study. Eur J Neurosci.

[28] Rudroff T, Workman CD, Fietsam AC, Kamholz J (2020). Response variability in transcranial direct current stimulation: why sex matters. Front Psychiatry..

[29] de Tommaso M, Invitto S, Ricci K, Lucchese V, Delussi M, Quattromini P (2014). Effects of anodal TDCS stimulation of left parietal cortex on visual spatial attention tasks in men and women across menstrual cycle. Neurosci Lett.

[30] Cahill L (2006). Why sex matters for neuroscience. Nat Rev Neurosci.

[31] Halpern DF, Tan U (2001). Stereotypes and steroids: using a psychobiosocial model to understand cognitive sex differences. Brain Cogn.

[32] Smith M, Keel J, Greenberg B, Adams L, Schmidt P, Rubinow D (1999). Menstrual cycle effects on cortical excitability. Neurology.

[33] Smith MJ, Adams LF, Schmidt PJ, Rubinow DR, Wassermann EM (2002). Effects of ovarian hormones on human cortical excitability. Ann Neurol.

[34] Pletzer B, Kronbichler M, Nuerk HC, Kerschbaum H (2014). Hormonal contraceptives masculinize brain activation patterns in the absence of behavioral changes in two numerical tasks. Brain Res.

[35] Chaieb L, Antal A, Paulus W (2008). Gender-specific modulation of short-term neuroplasticity in the visual cortex induced by transcranial direct current stimulation. Vis Neurosci.

[36] Kuo MF, Paulus W, Nitsche MA (2006). Sex differences in cortical neuroplasticity in humans. NeuroReport.

[37] León JJ, Sánchez-Kuhn A, Fernández-Martín P, Páez-Pérez MA, Thomas C, Datta A (2020). Transcranial direct current stimulation improves risky decision making in women but not in men: a sham-controlled study. Behav Brain Res.

[38] Martin AK, Huang J, Hunold A, Meinzer M (2017). Sex mediates the effects of high-definition transcranial direct current stimulation on “mind-reading”. Neuroscience.

[39] Adenzato M, Manenti R, Gobbi E, Enrici I, Rusich D, Cotelli M (2019). Aging, sex and cognitive theory of mind: a transcranial direct current stimulation study. Sci Rep.

[40] Tremblay S, Lepage JF, Latulipe-Loiselle A, Fregni F, Pascual-Leone A, Théoret H (2014). The uncertain outcome of prefrontal tDCS. Brain Stimulat.

[41] DeCasien AR, Guma E, Liu S, Raznahan A (2022). Sex differences in the human brain: a roadmap for more careful analysis and interpretation of a biological reality. Biol Sex Differ.

[42] Sommer A, Ecker L, Plewnia C (2021). Neural signatures of performance feedback in the paced auditory serial addition task (PASAT): an ERP study. Front Hum Neurosci.

[43] Oldfield RC (1971). The assessment and analysis of handedness: the Edinburgh inventory. Neuropsychologia.

[44] Schade S, Moliadze V, Paulus W, Antal A (2012). Modulating neuronal excitability in the motor cortex with tDCS shows moderate hemispheric asymmetry due to subjects’ handedness: a pilot study. Restor Neurol Neurosci.

[45] Rheinberg F, Vollmeyer R, Burns B (2001). QCM: a questionnaire to assess current motivation in learning situations. Diagnostica.

[46] Rosenberg M. Black and white self-esteem: the urban school child. American Sociological Association; 1971.

[47] Xu ML, Leung SO (2018). Effects of varying numbers of Likert scale points on factor structure of the Rosenberg Self-Esteem Scale. Asian J Soc Psychol.

[48] R Core Team. R: a language and environment for statistical computing. R Found Stat Comput. 2018; https://www.R-project.org/

[49] R Core Team. nlme: linear and nonlinear mixed effects models. R Found Stat Comput. 2018; https://CRAN.R-project.org/package=nlme

[50] Hughes J. reghelper: helper funtions for regression analysis. R Found Stat Comput. 2018. https://CRAN.R-project.org/package=reghelper

[51] Armonk, NY: IBM Corp. IBM SPSS Statistics for Windows, Version 24.0.0.1. 2016.

[52] Enders CK, Tofighi D (2007). Centering predictor variables in cross-sectional multilevel models: a new look at an old issue. Psychol Methods.

[53] Schuurman NK, Ferrer E, de Boer-Sonnenschein M, Hamaker EL (2016). How to compare cross-lagged associations in a multilevel autoregressive model. Psychol Methods.

[54] Nezlek JB. Multilevel modeling for psychologists. In: APA handbook of research methods in psychology, Vol 3: Data analysis and research publication. Washington, DC, US: American Psychological Association; 2012. p. 219–41. (APA handbooks in psychology®).

[55] Aguinis H, Gottfredson RK, Culpepper SA (2013). Best-practice recommendations for estimating cross-level interaction effects using multilevel modelling. J Manag.

[56] de Boer NS, Schluter RS, Daams JG, van der Werf YD, Goudriaan AE, van Holst RJ (2021). The effect of non-invasive brain stimulation on executive functioning in healthy controls: a systematic review and meta-analysis. Neurosci Biobehav Rev.

[57] Licata AE, Zhao Y, Herrmann O, Hillis AE, Desmond J, Onyike C (2023). Sex differences in effects of tDCS and language treatments on brain functional connectivity in primary progressive aphasia. NeuroImage Clin.

[58] Meiron O, Lavidor M (2013). Unilateral prefrontal direct current stimulation effects are modulated by working memory load and gender. Brain Stimulat.

[59] He X, Hu J, Qi Y, Turel O, Bechara A, He Q (2023). Sex modulates the effect of HD-tDCS over the prefrontal cortex on the Iowa Gambling Task. Brain Stimulat.

[60] Mosayebi Samani M, Agboada D, Jamil A, Kuo MF, Nitsche MA (2019). Titrating the neuroplastic effects of cathodal transcranial direct current stimulation (tDCS) over the primary motor cortex. Cortex.

[61] Weiss M, Lavidor M (2012). When less is more: evidence for a facilitative cathodal tDCS effect in attentional abilities. J Cogn Neurosci.

[62] Williams CM, Peyre H, Toro R, Ramus F (2021). Sex differences in the brain are not reduced to differences in body size. Neurosci Biobehav Rev.

[63] Eliot L, Ahmed A, Khan H, Patel J (2021). Dump the “dimorphism”: comprehensive synthesis of human brain studies reveals few male-female differences beyond size. Neurosci Biobehav Rev.

[64] Williams CM, Peyre H, Toro R, Ramus F (2021). Neuroanatomical norms in the UK Biobank: the impact of allometric scaling, sex, and age. Hum Brain Mapp.

[65] Joel D (2021). Beyond the binary: rethinking sex and the brain. Neurosci Biobehav Rev.

[66] Mylius V, Ayache SS, Ahdab R, Farhat WH, Zouari HG, Belke M (2013). Definition of DLPFC and M1 according to anatomical landmarks for navigated brain stimulation: Inter-rater reliability, accuracy, and influence of gender and age. Neuroimage.

[67] Luders E, Toga AW. Chapter 1—sex differences in brain anatomy. In: Savic I, editor. Progress in brain research. Elsevier; 2010. p. 2–12. (Sex differences in the human brain, their underpinnings and implications; vol. 186). https://www.sciencedirect.com/science/article/pii/B9780444536303000014. Accessed 18 Apr 2023. 10.1016/B978-0-444-53630-3.00017-8 21094881

[68] Luders E, Gaser C, Narr KL, Toga AW (2009). Why sex matters: brain size independent differences in gray matter distributions between men and women. J Neurosci.

[69] Butler T, Pan H, Imperato-McGinley J, Voyer D, Cunningham-Bussel AC, Cordero JJ (2007). A network approach to fMRI condition-dependent cognitive activation studies as applied to understanding sex differences. Clin Neurosci Res.

[70] Bangasser DA, Eck SR, Telenson AM, Salvatore M (2018). Sex differences in stress regulation of arousal and cognition. Physiol Behav.

[71] Koch K, Pauly K, Kellermann T, Seiferth NY, Reske M, Backes V (2007). Gender differences in the cognitive control of emotion: an fMRI study. Neuropsychologia.

[72] Mak AKY, Hu ZG, Zhang JXX, Xiao Z, Lee TMC (2009). Sex-related differences in neural activity during emotion regulation. Neuropsychologia.

[73] Weiss E, Siedentopf CM, Hofer A, Deisenhammer EA, Hoptman MJ, Kremser C (2003). Sex differences in brain activation pattern during a visuospatial cognitive task: a functional magnetic resonance imaging study in healthy volunteers. Neurosci Lett.

[74] Ruf SP, Fallgatter AJ, Plewnia C. Augmentation of working memory training by transcranial direct current stimulation (tDCS). Sci Rep. 2017;7(1). http://www.nature.com/articles/s41598-017-01055-1. Accessed 1 Aug 2018. 10.1038/s41598-017-01055-1 PMC5430723 28432349

[75] Grissom NM, Reyes TM (2018). Let’s call the whole thing off: evaluating gender and sex differences in executive function. Neuropsychopharmacology.

[76] Kuhn L, Noack H, Wagels L, Prothmann A, Schulik A, Aydin E (2023). Sex-dependent multimodal response profiles to psychosocial stress. Cereb Cortex N Y N 1991..

[77] Kogler L, Seidel EM, Metzler H, Thaler H, Boubela RN, Pruessner JC (2017). Impact of self-esteem and sex on stress reactions. Sci Rep.

[78] Tombaugh TN (2006). A comprehensive review of the paced auditory serial addition test (PASAT). Arch Clin Neuropsychol.

[79] Santesso DL, Dzyundzyak A, Segalowitz SJ (2011). Age, sex and individual differences in punishment sensitivity: factors influencing the feedback-related negativity. Psychophysiology.

[80] Esmaeilpour Z, Shereen AD, Ghobadi-Azbari P, Datta A, Woods AJ, Ironside M (2020). Methodology for tDCS integration with fMRI. Hum Brain Mapp.

[81] Li LM, Violante IR, Leech R, Ross E, Hampshire A, Opitz A (2019). Brain state and polarity dependent modulation of brain networks by transcranial direct current stimulation. Hum Brain Mapp.

[82] Mandrick K, Peysakhovich V, Rémy F, Lepron E, Causse M (2016). Neural and psychophysiological correlates of human performance under stress and high mental workload. Biol Psychol.

[83] Indahlastari A, Albizu A, O’Shea A, Forbes MA, Nissim NR, Kraft JN (2020). Modeling transcranial electrical stimulation in the aging brain. Brain Stimulat.

[84] Goldfarb EV, Rosenberg MD, Seo D, Constable RT, Sinha R (2020). Hippocampal seed connectome-based modeling predicts the feeling of stress. Nat Commun.

[85] Goldfarb EV, Seo D, Sinha R (2019). Sex differences in neural stress responses and correlation with subjective stress and stress regulation. Neurobiol Stress.

[86] Finocchi C, Ferrari M (2011). Female reproductive steroids and neuronal excitability. Neurol Sci.

[87] Mac Giolla E, Kajonius PJ (2019). Sex differences in personality are larger in gender equal countries: replicating and extending a surprising finding. Int J Psychol.

