# Supplementary material for: Sex matters for the enhancement of cognitive training with transcranial direct current stimulation (tDCS)
Source: Biol Sex Differ. 2023 Nov 2;14:78. doi: 10.1186/s13293-023-00561-4 (PMC10623760; doi:10.1186/s13293-023-00561-4)
Supplement: Supplementary file 1 — Additional file 1: Table S1. Training performance as measured by the number of correct trials for all groups. Shown are mean of correct trials and standard deviations in parentheses. Table S2. Results from the linear mixed model for all males and females pooled, regardless of tDCS setting. All calculations use the female group (fP) as a reference. Number of subjects: N = 162. Table S3. Results from the linear mixed model for groups organised by tDCS polarity (sham). All calculations use the female group (fS) as a reference. Number of subjects: N = 43. Table S4. Results from the linear mixed model for groups organised by tDCS polarity (anodal). All calculations use the female group (fA) as a reference. Number of subjects: N = 60. Table S5. Results from the linear mixed model for groups organised by tDCS polarity (cathodal). All calculations use the female group (fC) as a reference. Number of subjects: N = 59. Table S6. Results from the linear mixed model from the male group divided by tDCS polarity. All calculations use the sham group (mS) as a reference. Number of subjects: N = 35. Table S7. Results from the linear mixed model from the female group divided by tDCS polarity. All calculations use the sham group (fS) as a reference. Number of subjects: N = 127. [file 13293_2023_561_MOESM1_ESM.docx]

**Additional Tables**

**Table S1**. Training performance as measured by the number of correct trials for all groups. Shown are mean of correct trials and standard deviations in parentheses.

| Group | **Session** | | | | | | | | |
| --- | --- | --- | --- | --- | --- | --- | --- | --- | --- |
|  | **1** | **2** | **3** | **4** | **5** | **6** | **7** | **8** | **9** |
| m_P_ | 129.11 (5.64) | 165.23 (6.70) | 185.8 (7.36) | 202.94 (7.14) | 212.25 (7.54) | 222.29 (7.41) | 222.29 (7.92) | 225.37 (8.62) | 225.46 (7.86) |
| f_P_ | 120.61 (2.93) | 160.07 (3.63) | 186.48 (3.89) | 203.61 (3.92) | 217.66 (3.78) | 229.22 (3.56) | 234.97 (3.65) | 238.29 (3.47) | 232.18  (3.62) |
| m_S_ | 134.46 (12.44) | 167.64 (15.43) | 193 (15.69) | 204.273 (14.6) | 216.82  (14,65) | 221 (15.22) | 223.09 (17.60) | 229 (17.89) | 220.36 (17.87) |
| f_S_ | 113.25 (5.44) | 153.28 (7.79) | 179.13 (9.07) | 193.84 (8.24) | 205.156 (8.73) | 216.19 (8.76) | 222.75 (8.72) | 229.94 (8.79) | 219.25 (8.53) |
| m_A_ | 137.55 (8.18) | 173 (8.55) | 189.36 (12.93) | 212.64 (13.36) | 215.64 (12.97) | 227.18 (13.38) | 230.91 (10.86) | 234.36 (13.25) | 230.46 (13.59) |
| f_A_ | 119.06 (4.98) | 157.25 (5.81) | 185.29 (6.16) | 204.74 (6.33) | 219.67 (5.84) | 231.47 (5.32) | 240.45 (5.73) | 240.45 (5.30) | 234.82 (5.71) |
| m_C_ | 117.46 (8.25) | 156.62 (10.61) | 176.69 (10.50) | 193.62 (9.89) | 205.54 (11.61) | 219.23 (11.25) | 214.31 (12.89) | 214.69 (14.17) | 225.54  (10.57) |
| f_C_ | 127.39 (4.71) | 167.80 (5.68) | 192.83 (5.68) | 209.20 (6.22) | 224.22 (5.62) | 235.89  (5.02) | 237.63  (5.10) | 242.09 (4.67) | 238.66 (4.98) |

**Table S2**. Results from the linear mixed model for all males and females pooled, regardless of tDCS setting. All calculations use the female group (f_P_) as a reference. Number of subjects: *N* = 162.

| Factor | Δ *n_corr_* | SEM | *df* | *t* | *p* |
| --- | --- | --- | --- | --- | --- |
| Time | 14.748 | 0.514 | 807 | 28.707 | <0.0001* |
| Pre-training | 1.108 | 0.047 | 159 | 23.406 | <0.0001* |
| m_P_ | 1.428 | 4.791 | 159 | 0.298 | 0.7660 |
| m_P_:time | -3.204 | 1.105 | 807 | -2.900 | 0.0038* |

**Table S3**. Results from the linear mixed model for groups organised by tDCS polarity (sham). All calculations use the female group (f_S_) as a reference. Number of subjects: *N* = 43.

| Factor | Δ *n_corr_* | SEM | *df* | *t* | *p* |
| --- | --- | --- | --- | --- | --- |
| Time | 13.424 | 1.095 | 213 | 12.265 | <0.0001* |
| Pre-training | 1.286 | 0.101 | 40 | 12.750 | <0.0001* |
| m_S_ | -5.676 | 8.932 | 40 | -0.635 | 0.5288 |
| m_S_:time | -2.744 | 2.164 | 213 | -1.268 | 0.2063 |

**Table S4**. Results from the linear mixed model for groups organised by tDCS polarity (anodal). All calculations use the female group (f_A_) as a reference. Number of subjects: *N* = 60.

| Factor | Δ *n_corr_* | SEM | *df* | *t* | *p* |
| --- | --- | --- | --- | --- | --- |
| Time | 16.228 | 0.730 | 297 | 22.227 | <0.0001* |
| Pre-training | 1.055 | 0.078 | 57 | 13.569 | <0.0001* |
| m_A_ | 2.876 | 7.899 | 57 | 0.364 | 0.7172 |
| m_A_:time | -4.628 | 1.704 | 297 | -2.715 | 0.0070* |

**Table S5**. Results from the linear mixed model for groups organised by tDCS polarity (cathodal). All calculations use the female group (f_C_) as a reference. Number of subjects: *N* = 59.

| Factor | Δ *n_corr_* | SEM | *df* | *t* | *p* |
| --- | --- | --- | --- | --- | --- |
| Time | 14.096 | 0.890 | 293 | 15.830 | <0.0001* |
| Pre-training | 1.036 | 0.074 | 56 | 13.982 | <0.0001* |
| m_C_ | 1.759 | 8.566 | 56 | 0.205 | 0.8381 |
| m_C_:time | -1.867 | 1.897 | 293 | -0.984 | 0.3258 |

**Table S6**. Results from the linear mixed model from the male group divided by tDCS polarity. All calculations use the sham group (m_S_) as a reference. Number of subjects: *N* = 35.

| Factor | Δ *n_corr_* | SEM | *df* | *t* | *p* |
| --- | --- | --- | --- | --- | --- |
| Time | 10.681 | 1.618 | 172 | 6.602 | <0.0001* |
| Pre-training | 1.134 | 0.096 | 31 | 11.782 | <0.0001* |
| m_A_ | -3.823 | 10.170 | 31 | -0.376 | 0.7095 |
| m_C_ | 2.327 | 9.903 | 31 | 0.235 | 0.8158 |
| m_A_:time | 0.919 | 2.288 | 172 | 0.402 | 0.6882 |
| m_C_:time | 1.548 | 2.198 | 172 | 0.704 | 0.4822 |

**Table S7**. Results from the linear mixed model from the female group divided by tDCS polarity. All calculations use the sham group (f_S_) as a reference. Number of subjects: *N* = 127.

| Factor | Δ *n_corr_* | SEM | *df* | *t* | *p* |
| --- | --- | --- | --- | --- | --- |
| Time | 13.424 | 1.034 | 631 | 12.982 | <0.0001* |
| Pre-training | 1.115 | 0.056 | 123 | 19.811 | <0.0001* |
| f_A_ | -7.587 | 5.801 | 123 | -1.308 | 0.1933 |
| f_C_ | -2.583 | 5.917 | 123 | -0.436 | 0.6632 |
| f_A_:time | 2.803 | 1.330 | 631 | 2.108 | 0.0354* |
| f_C_:time | 0.672 | 1.347 | 631 | 0.499 | 0.618 |
